# Comparative transcriptomic profiling of peripheral efferent and afferent nerve fibres at different developmental stages in mice

**DOI:** 10.1038/s41598-018-30463-0

**Published:** 2018-08-10

**Authors:** Hongkui Wang, Youlang Zhou, Meng Cong, Li Zhang, Xiaosong Gu, Xin Tang

**Affiliations:** 1 Key Laboratory of Neuroregeneration of Jiangsu and Ministry of Education, Co-Innovation Center of Neuroregeneration, Nantong University, Nantong, JS 226001 China; 2 The Hand Surgery Research Center, Department of Hand Surgery, Affiliated Hospital of Nantong University, Nantong, JS 226001 China

## Abstract

Peripheral nerve injury impairs motor and sensory function in humans, and its functional recovery largely depends on the axonal outgrowth required for the accurate reinnervation of appropriate targets. To better understand how motor and sensory nerve fibres select their terminal pathways, an unbiased cDNA microarray analysis was conducted to examine differential gene expression patterns in peripheral efferent and afferent fibres at different developmental stages in mice. Gene ontology (GO) and Kyoto Enrichment of Genes and Genomes (KEGG) analyses revealed common and distinct features of enrichment for differentially expressed genes during motor and sensory nerve fibre development. Ingenuity Pathway Analysis (IPA) further indicated that the key differentially expressed genes were associated with trans-synaptic neurexin-neuroligin signalling components and a variety of gamma-aminobutyric acid (GABA) receptors. The aim of this study was to generate a framework of gene networks regulated during motor and sensory neuron differentiation/maturation. These data may provide new clues regarding the underlying cellular and molecular mechanisms that determine the intrinsic capacity of neurons to regenerate after peripheral nerve injury. Our findings may thus facilitate further development of a potential intervention to manipulate the therapeutic efficiency of peripheral nerve repair in the clinic.

## Introduction

Injured axons in the adult mammalian peripheral nervous system (PNS), unlike those in the central nervous system (CNS), were considered to have the intrinsic ability to initiate regeneration in the appropriate context^1–4^. However, numerous studies have indicated that this endogenous regenerative capacity is still limited since functional recovery is usually poor in the context of complete axonal transection with relatively large gap distances^5^. Peripheral nerve injury has a deleterious impact on daily life, as it significantly impairs motor and sensory function in patients. Therefore, a better understanding of how efferent and afferent nerve fibres select their terminal pathways, and further, how proximal axonal sprouts reach and reinnervate their topographically appropriate targets^6^, is of fundamental interest to both clinical and basic neuroscientists^7–9^.

Generally, functional recovery after peripheral nerve injury largely depends on the accuracy of axonal pathfinding and the efficiency of reinnervation of distal target organs^10,11^. A central issue in neural regeneration is the manner in which various neurons reestablish their distinct and predictable phenotypes by forming functionally appropriate synaptic connections^10^. Previous studies have indicated that regenerating motor axons are guided through the motor pathways to reestablish nerve-muscle contact in a process termed ‘preferential motor reinnervation’^12,13^. However, the molecular mechanism underlying this phenomenon is not yet fully understood^14,15^.

The sciatic nerve is the largest single nerve in humans. The rodent sciatic nerve injury model is the most commonly used model for studying peripheral nerve regeneration. The spinal segments associated with the sciatic nerve correspond to the lumbar dorsal root ganglia (L4-L6 DRGs)^16,17^. Efferent nerve fibres are axons originating from motor neurons, while afferent nerve fibres are processes extending from sensory neurons. In the spinal cord, motor neuron cell bodies are present in the ventral horn of the grey matter, and receive inputs from interneurons transmitting signals via sensory integration feedback^18^. Efferent nerve fibres conduct signals away from the CNS to their target peripheral organs, and form neuromuscular junctions with innervated muscles. Unlike motor neuron somas, sensory neuron somas are located in the DRGs of the PNS. Axonal processes from groups of afferent neuron somas extend in two opposite directions: the peripheral branch runs into the spinal nerve to innervate the peripheral target organs, while the central branch runs to the dorsal horn of the spinal cord, ultimately conveying sensory information from the PNS to the CNS^19^. During the development of selective sensory-motor circuits, fundamental biological processes, such as axon guidance, selective target reinnervation, and synaptic plasticity in neural networks, must be regulated properly to establish the appropriate neural circuits^20^.

In the present study, an unbiased cDNA microarray analysis was performed to examine differential gene expression patterns in both efferent and afferent nerve fibres at different developmental stages in mice. The detection window was from the postnatal day 0 (P0) to 5 weeks (5 W), as the de novo axonal sprouting and synaptic formation and remodelling during the regenerative processes is analogous with those in the early developmental stages^2,21^. The aim of this study was to provide a framework for the integrated analysis of gene regulatory networks during peripheral motor and sensory neuronal differentiation/maturation. We also aimed to further elucidate the biological processes and molecular mechanisms underlying the activation of the intrinsic neuronal regenerative capacity after peripheral nerve injury. These data provide new insights for the development of potential interventions designed to manipulate the therapeutic efficiency of peripheral nerve repair in the clinic.

## Results

### Tissue Collection Process and Microarray Data Quality Assessment

**Figure 1:**
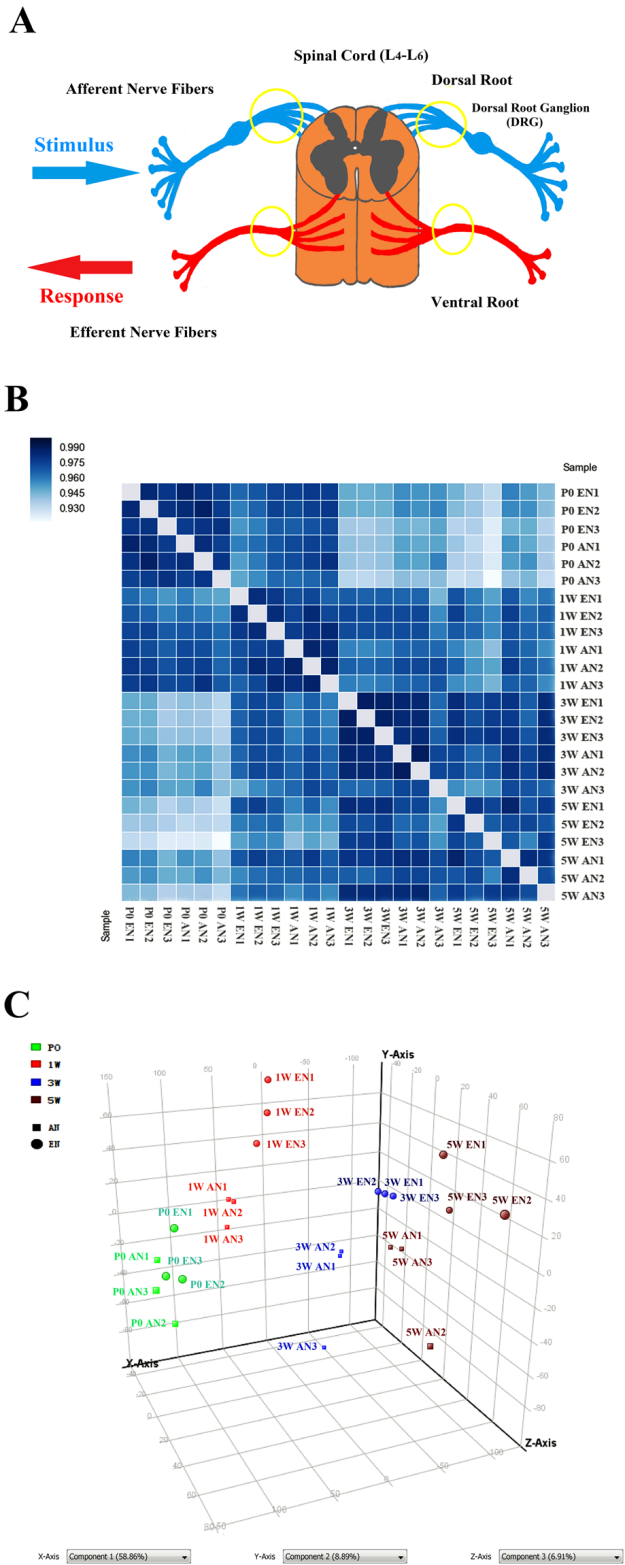
Schematic diagram illustrating tissue collection and microarray data quality assessment. Tissues were dissected from the areas highlighted using circles in the diagram, which contain peripheral nerve fibres extending from the spinal cord (**A**). Sample correlation matrices (**B**) and three-dimensional principal component analysis plot (**C**) demonstrating a reasonable clustering of the 24 samples (8 groups in triplicate).

We performed cDNA microarray analysis using RNA samples extracted from efferent and afferent nerve fibres originating from the L4–L6 segments of the spinal cord. The dissection range is illustrated using circles in the spinal cord diagram (Fig. 1A). At different developmental stages (P0, 1 week [1 W], 3 weeks [3 W], and 5 weeks [5 W]), the efferent and afferent nerve fibres (also known as the ventral and dorsal root fibres, respectively) were carefully dissected and collected using an anatomy microscope.

The sample correlation matrices indicated that all the 24 samples (8 groups in triplicate) could be approximately clustered into four sets corresponding to the four experimental time points (Fig. 1B). Additionally, the three-dimensional principal component analysis (PCA) plot indicates that the samples could be generally classified based on the time points and the category of efferent vs. afferent nerve fibre (Fig. 1C).

### General Overview of Differentially Expressed Genes in Efferent and Afferent Nerve Fibres at Different Developmental Stages

**Figure 2:**
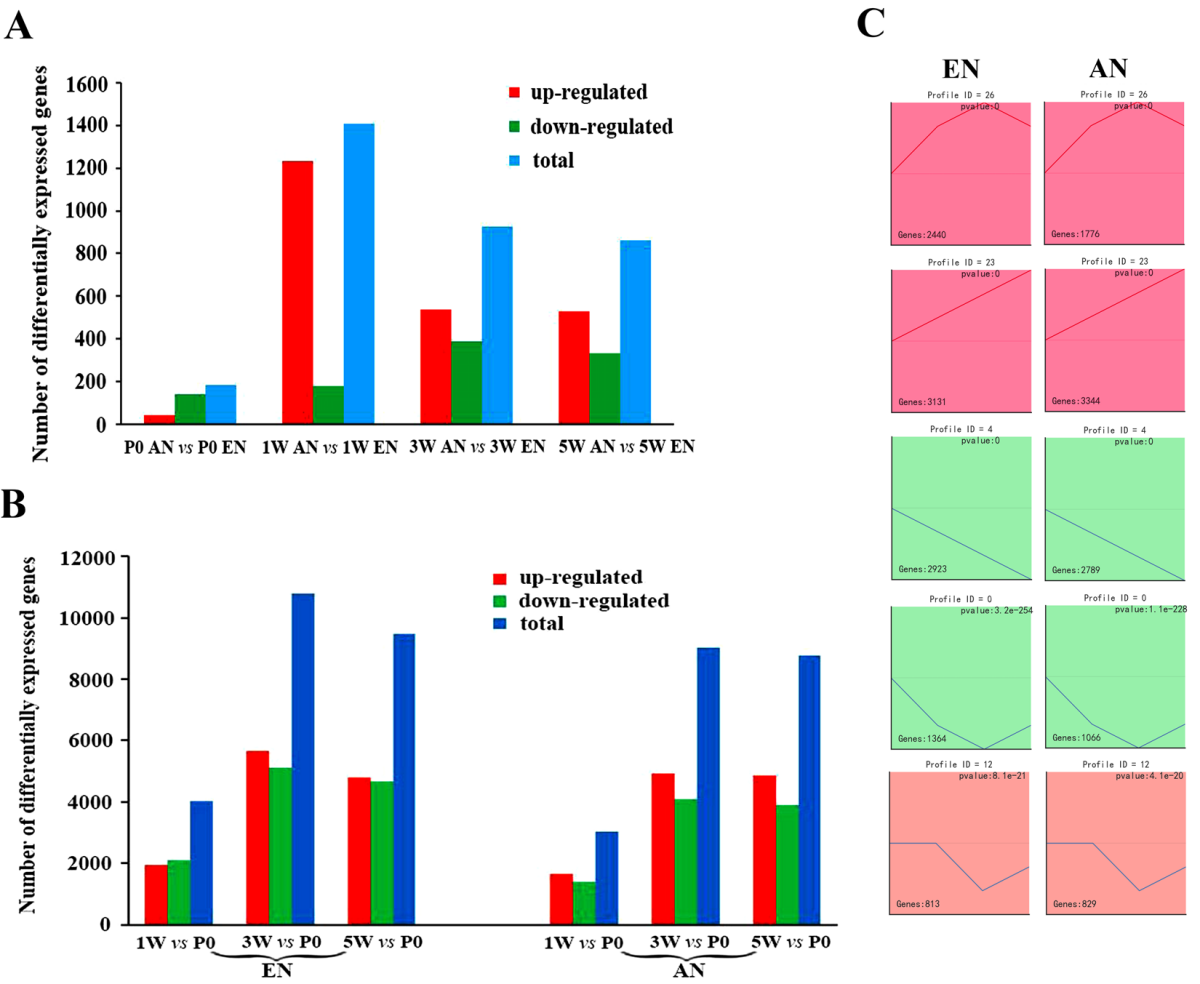
General overview of differentially expressed genes in efferent and afferent nerve fibres during development. Histogram (**A**) showing a comparison of differentially expressed genes between efferent and afferent nerve fibres at different developmental stages (postnatal day 0 [P0], 1 week [1 W], 3 weeks [3 W], and 5 weeks [5 W]). Histogram (**B**) showing the number of genes differentially expressed in the efferent or afferent nerve fibres throughout development (up-regulated in red, down-regulated in green, and total number in blue). Five distinct expression patterns in the efferent and afferent nerve fibres during development are shown for comparison (**C**).

The histograms in Fig. 2A show the results of the differential expression analysis in efferent vs. afferent nerve fibres at different time points. These data indicate that the maximum differential expression occurred at 1 W. This implies that the time period from P0 to 1 W is the time window for motor and sensory neuronal cell fate determination. The differential gene expression declined with time during the postnatal period from 1 W through 5 W.

Figure 2B indicates that very large numbers of genes were differentially expressed in the efferent and afferent nerve fibres throughout development. It is noteworthy that both types of nerve fibres displayed the maximum number of differentially expressed genes at the 3 W time point.

The temporal expression profiles of the significantly differentially expressed genes in efferent and afferent nerve fibres were visualized using Short Time-series Expression Miner (STEM) software. A comparison of the five distinct expression patterns of the genes differentially expressed between the efferent and afferent nerve fibres is presented in Fig. 2C. The total numbers of differentially expressed genes in the efferent and afferent nerve fibres were 12,897 and 11,817, respectively. Of these genes, 2,440 genes in efferent nerve fibres and 1,776 genes in afferent nerve fibres were first up-regulated and reached a peak in expression at 3 W. The expression levels of these genes then recovered at 5 W following an inverted U-shaped curve. Additionally, 3,131 genes in the efferent nerve fibres and 3,344 genes in afferent nerve fibres were linearly up-regulated at all experimental time points; 2,923 genes in the efferent nerve fibres and 2,789 genes in afferent nerve fibres were linearly down-regulated during development. Moreover, 1,364 genes in the efferent nerve fibres and 1,066 genes in afferent nerve fibres were first down-regulated from P0 to 3 W and then recovered at 5 W following a U-shaped curve. Lastly, 813 genes in the efferent nerve fibres and 829 genes in afferent nerve fibres had unchanged expression levels at P0 and 1 W, were down-regulated at 3 W, and were then up-regulated at 5 W (Fig. 2C and Table S2).

### Hierarchical Clustering Analysis of Differentially Expressed Genes

**Figure 3:**
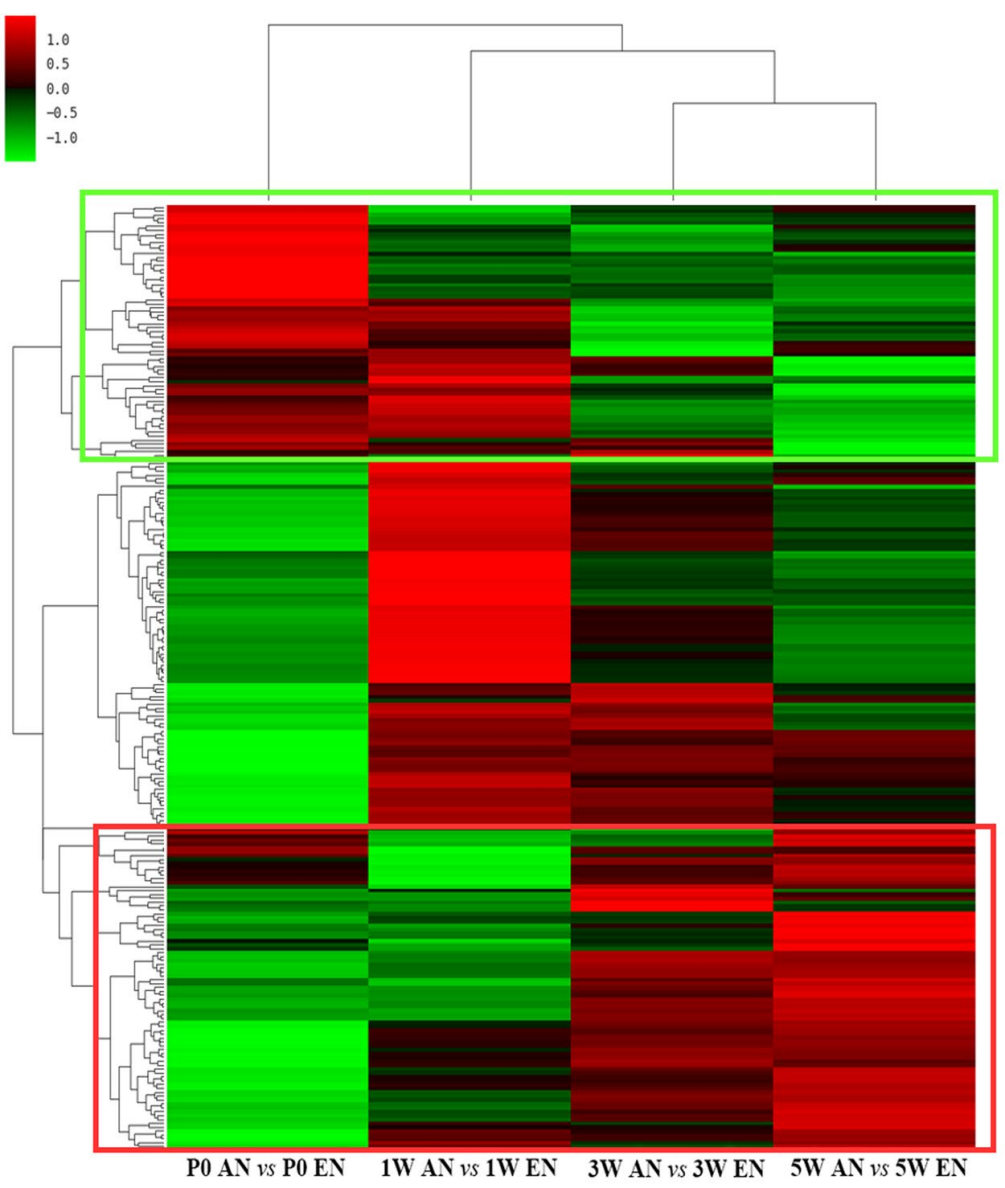
Heat map displaying the hierarchical clustering of differentially expressed genes in efferent and afferent nerve fibres during development. The relative expression level of each gene is indicated by colour intensity. Genes that were generally down-regulated are highlighted in green rectangles, while genes that were generally up-regulated are highlighted in red rectangles.

Hierarchical clustering analysis was performed on differentially expressed genes in efferent and afferent nerve fibres at each experimental time point. The results were visualized using heat map images. The heat maps indicated that nearly 1/4^th^ of the differentially expressed genes in the efferent and afferent nerve fibres were first up-regulated and then down-regulated, while a little less than 1/3^rd^ of the differentially expressed genes were first down-regulated and then up-regulated at all developmental stages. The gene expression patterns in the motor and sensory nerve fibres during development are shown in Fig. 3. All the differentially expressed genes involved in efferent and afferent nerve fibre development are listed in Table S3.

### GO Analysis of Biological Processes, Cellular Components, and Molecular Functions Involved in Efferent and Afferent Nerve Fibre Development

**Figure 4:**
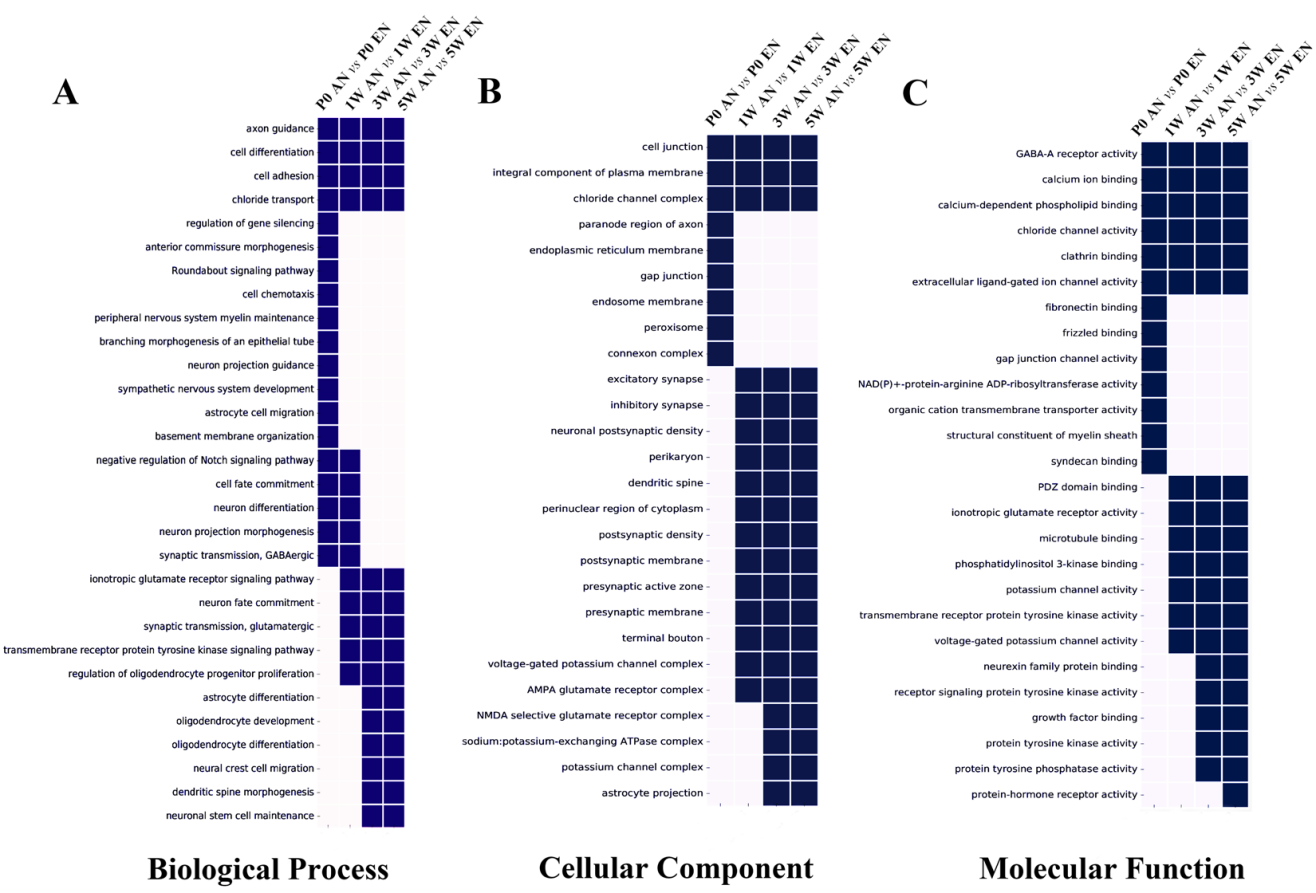
Enriched gene ontology analysis of differentially expressed genes. Comparison of genes differentially expressed between efferent and afferent nerve fibres at different developmental stages (postnatal day 0 [P0], 1 week [1 W], 3 weeks [3 W], and 5 weeks [5 W]), categorized using Biological Processes (**A**), Cellular Components (**B**), and Molecular Functions (**C**). Square with *p* < 0.05 are labelled in blue, while those with *p* > 0.05 are labelled in white.

In order to analyse the patterns of differential gene expression in the efferent and afferent nerve fibres during the different developmental stages, the GO analysis database integrated with DAVID’s toolkit was used to identify the most significant differentially expressed genes associated with neuronal development and outgrowth. There genes were categorised based on the following defined terms: Biological Processes, Cellular Components, and Molecular Functions (Fig. 4).

The enriched subcategories under Biological Processes in the efferent and afferent nerve fibres during development are presented in Fig. 4A. Among them, four biological processes (axon guidance, cell differentiation, cell adhesion, and chloride transport) were differentially expressed in the efferent and afferent nerve fibres at all time points. It is noteworthy that the anterior commissure morphogenesis, roundabout signalling pathway, cell chemotaxis, and neuron projection guidance categories only exhibited significant differential expression at P0. In these categories, there were no statistical differences between the two types of nerve fibres after P0.

In the Cellular Components category, the GO analysis indicated that the excitatory synapse and inhibitory synapse subcategories had no statistically significant differences between the efferent and afferent nerve fibres at P0, although they had significant differential expression starting at 1 W. In addition, the differential expression patterns were similar to those in the presynaptic and postsynaptic membrane subcategories (Fig. 4B).

The enrichment of Molecular Function in the different developmental stages is summarized in Fig. 4C. Intriguingly, gamma-aminobutyric acid A (GABAA) receptor activity, calcium ion binding, chloride channel activity, clathrin binding, and extracellular ligand-gated ion channel activity subcategories were found to have differential expression at all four experimental time points. In contrast, the ionotropic glutamate receptor activity and transmembrane receptor protein tyrosine kinase activity subcategories displayed no statistically significant differences at P0, but had significant differential expression at 1 W and thereafter.

### Critical Signalling Pathways Involved in Efferent and Afferent Nerve Fibre Development

**Figure 5:**
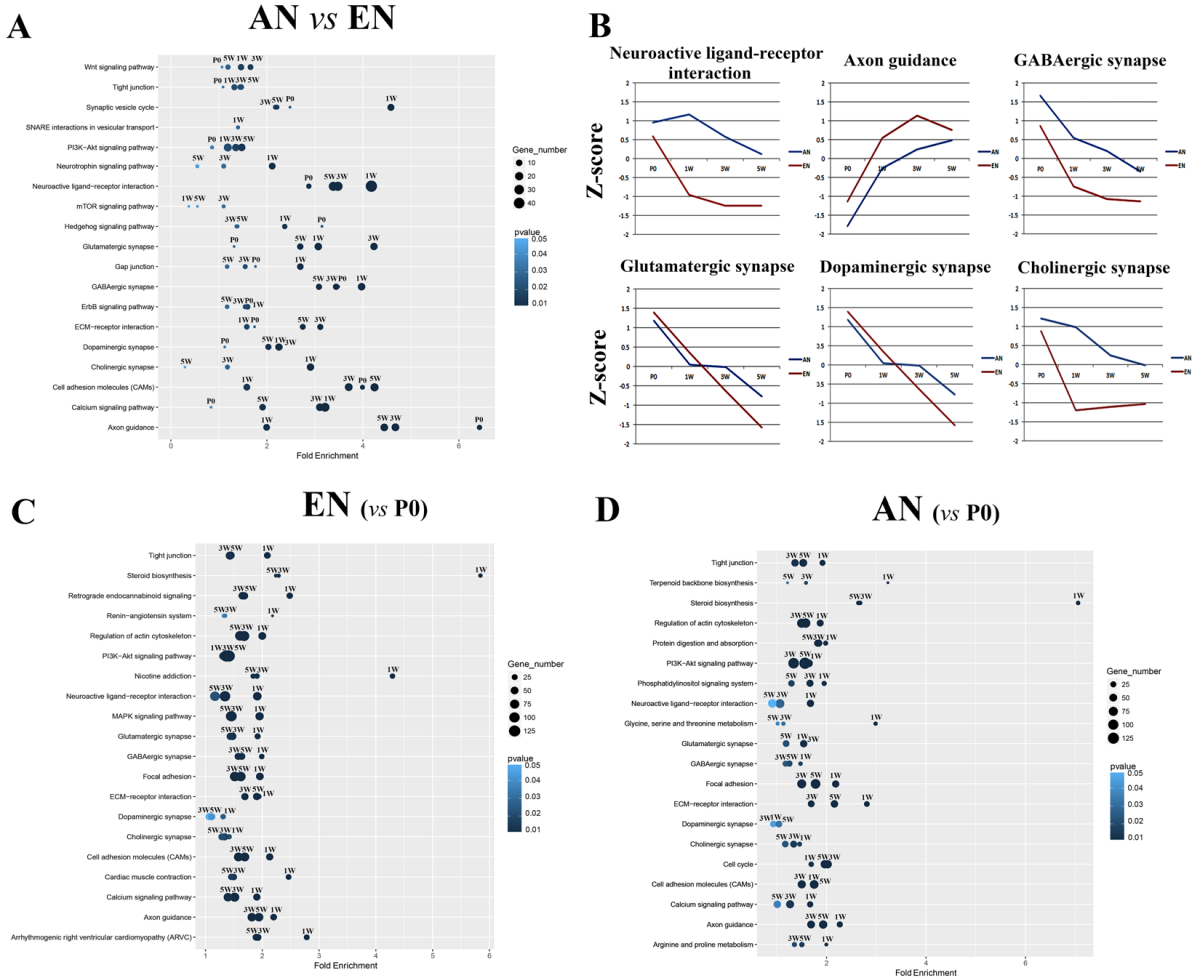
Enriched KEGG pathways for the differentially expressed genes. Comparison of genes differentially expressed between efferent and afferent nerve fibres (**A**) in different developmental processes, and Z-scores for the six critical Kyoto Enrichment of Genes and Genomes (KEGG) pathways (**B**). The differentially expressed genes in efferent (**C**) and afferent (**D**) nerve fibres at postnatal developmental stages (1 week [1 W], 3 weeks [3 W], and 5 weeks [5 W]) were compared with those at postnatal day 0 (P0). The colour intensity of the dots represents the *p* value, and the size of the dots represents the ratio of differentially expressed genes in the specific KEGG pathway.

The GO analysis provided overall insight into the cellular and molecular regulation of motor and sensory neuronal cell fate determination. Kyoto Enrichment of Genes and Genomes (KEGG) analysis was further performed to identify critical signalling pathways during motor and sensory neuronal differentiation/maturation (Fig. 5). It is noteworthy that two signalling pathways, namely axon guidance and hedgehog signalling, were significantly differentially expressed between the efferent and afferent nerve fibres during development as early as P0. In contrast, significant differential expression of proteins involved in SNARE interactions during vesicular transport in the efferent and afferent nerve fibres only occurred at 1 W. In addition to these pathways, synaptic plasticity-associated signalling pathways and proteins involved in GABAergic and cholinergic synapses were significantly differentially expressed at 1 W. In contrast, glutamatergic and dopaminergic synapses were significantly differentially expressed at 3 W (Fig. 5A).

The Z-scores calculated from the average expression profiles of the differentially expressed genes involved in the six most critical signalling pathways during development are listed in Fig. 5B. The Z-score curves indicate that the changes in the axon guidance and GABAergic synapse pathways at different developmental stages were relatively similar between the efferent and afferent nerve fibres. In contrast, the expression changes for mRNAs involved in neuroactive ligand-receptor interactions, cholinergic synapses, glutamatergic synapses, and dopaminergic synapse pathways were different between the two types of nerve fibres. This implies that the fundamental patterns of gene expression inherent in motor and sensory neuronal cell fate determination have distinct features.

Although the differential expression signatures in the efferent and afferent nerve fibres during development had common characteristics, there were demonstrable differences between the two types of nerve fibres. Such differences were observed in the glutamatergic and dopaminergic synapse pathways. For instance, the significant differential expression of proteins in glutamatergic synapses was first detected at 1 W in efferent nerve fibres. In contrast, this distinctive change in expression was first observed at 3 W in the afferent nerve fibres. The significant differential expression of proteins at dopaminergic synapses was first detected at 1 W in efferent nerve fibres, and at 5 W in the afferent nerve fibres (Fig. 5C,D).

### Cascade Regulation of Differentially Expressed Genes Involved in Efferent and Afferent Nerve Fibre Development

**Figure 6:**
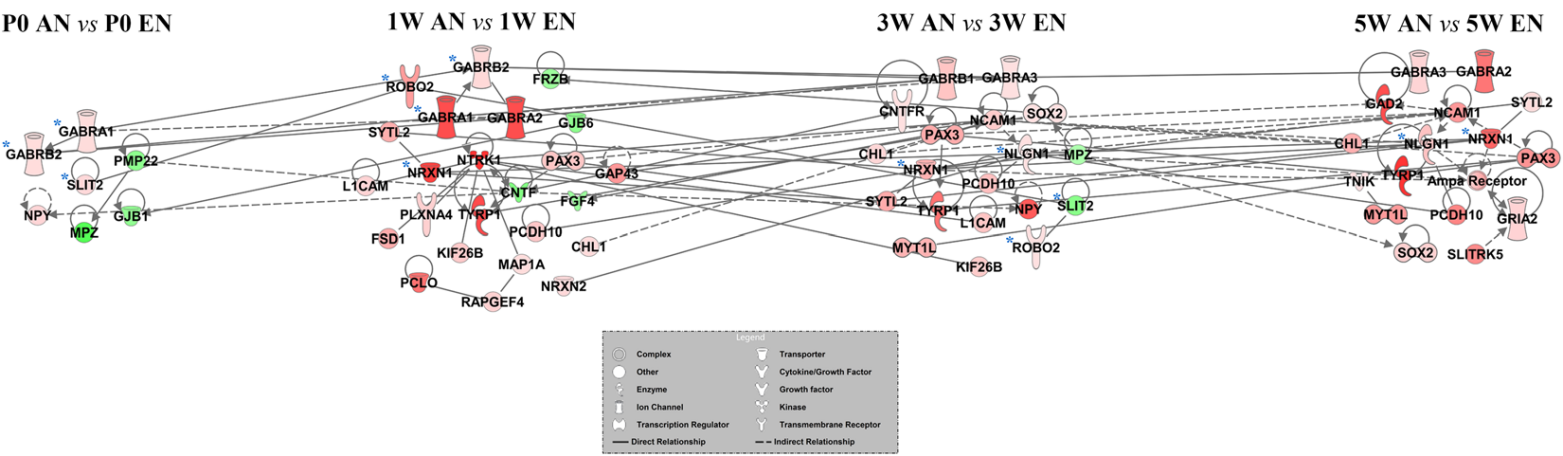
Cascade regulation of genes differentially expressed between efferent and afferent nerve fibres during development. The regulatory network exhibits the comparison of genes differentially expressed between efferent and afferent nerve fibres at different developmental stages (postnatal day 0 [P0], 1 week [1 W], 3 weeks [3 W], and 5 weeks [5 W]). The up-regulated genes are shown in red, while the down-regulated genes are shown in green, and the colour intensity indicates the fold change in differential expression. The key genes are highlighted with blue asterisks.

To further identify key genes differentially expressed between the efferent and afferent nerve fibres at each developmental time point, gene network diagrams were created using the IPA database (Fig. 6). We found that *Gabra1*, *Gabrb2*, and *Slit2* were differentially expressed between efferent and afferent nerve fibres as early as P0, and that multiple types of GABA receptors (GABARs) were consistently differentially expressed at later developmental stages. For instance, *Gabra1*, *Gabra2*, and *Gabrb2* were found to be differentially expressed at 1 W; *Gabra3* and *Gabrb1* were still differentially expressed at 3 W; and *Gabra2* and *Gabra3* were consistently differentially expressed between the efferent and afferent nerve fibres during development. Intriguingly, the neurexin family members *Nrxn1* and *Nrxn2* were significantly differentially expressed between the efferent and afferent nerve fibres at 1 W, and *Nrxn1* was differentially expressed even at later developmental stages. *Nlgn1*, which encodes neuroligin 1, the post-synaptic binding partner of neurexin, was differentially expressed between the efferent and afferent nerve fibres at 3 W and 5 W. This suggests that the developmental signature of the neuroligins was different from that of the neurexins during efferent and afferent nerve fibre development.

### Dynamic Changes in Differentially Expressed Genes Associated with Motor and Sensory Nerve Fibre Development

**Figure 7:**
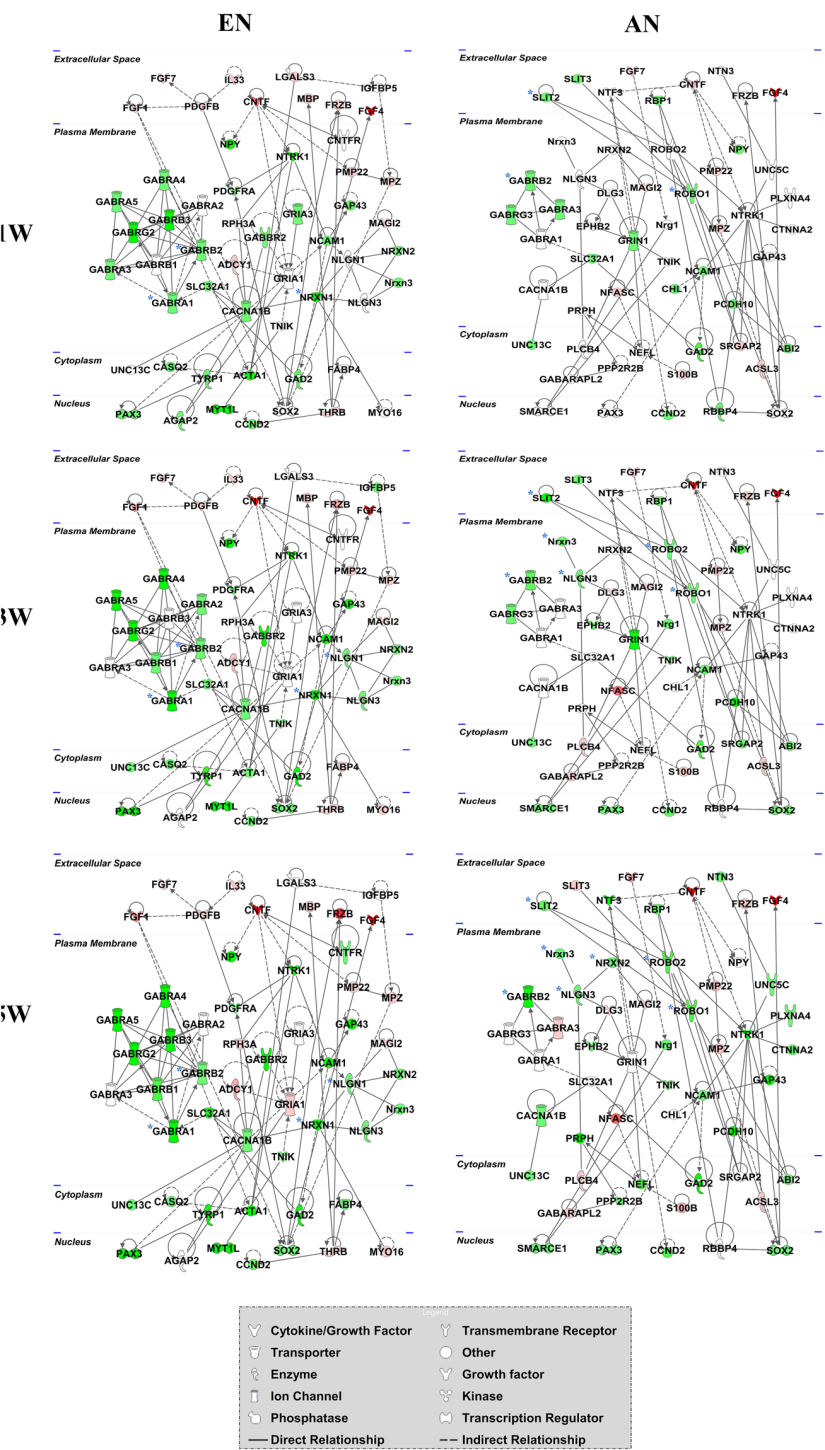
Dynamic changes in differentially expressed genes associated with efferent and afferent nerve fibre development. The gene network diagrams show the interactions and correlations of differentially expressed genes during efferent and afferent nerve fibre development at different postnatal stages (1 week [1 W], 3 weeks [3 W], and 5 weeks [5 W], when compared with postnatal day 0 [P0]). Red colour indicates the fold change in up-regulation, while the green colour indicates the fold change in down-regulation. Colour intensity indicates the relative fold change values. The key genes are highlighted with blue asterisks.

The regulatory networks for the genes differentially expressed during motor and sensory nerve fibre development were further analysed using the IPA database. The gene network diagram suggests that the differentially expressed genes were in highly correlated clusters in well-regulated networks during development (Fig. 7). During the motor nerve fibre development, *Gabrg2*, *Gabrb2*, and *Nrxn1* were dramatically downregulated at 1 W when compared with P0. This downregulation was sustained thereafter. Notably, a larger number of GABA receptors, such as *Gabra1*, *Gabra4*, and *Gabra5*, were further downregulated during motor nerve fibre development from 3 W to 5 W. *Nlgn1* was involved in motor nerve fibre development from 3 W to 5 W. In comparison, the slit guidance ligand (SLIT) family members *Slit2* and *Slit3* were downregulated in the extracellular space in sensory nerve fibres at 1 W when compared with P0. These mRNAs interacted with roundabout homologue 1 (ROBO1), which was downregulated in the plasma membrane. In addition, GABAR family members, such as *Gabra3*, *Gabrb2*, and *Gabrg3*, were down-regulated in sensory nerve fibres at 1 W when compared with P0. However, changes in the expression levels of these proteins in sensory nerve fibres were not as obvious as those observed in motor nerve fibres during development. For instance, the *Gabrb2* was dramatically downregulated together with *Nrxn3* at 3 W and 5 W, while *Gabra3* was only upregulated at 5 W. Furthermore, *Nrxn2* and *Nrxn3*, but not *Nrxn1*, were downregulated together with *Nlgn3*, which is involved in sensory nerve fibre development.

### RT-qPCR Validation of Key Genes Associated with Motor and Sensory Nerve Fibre Development

**Figure 8:**
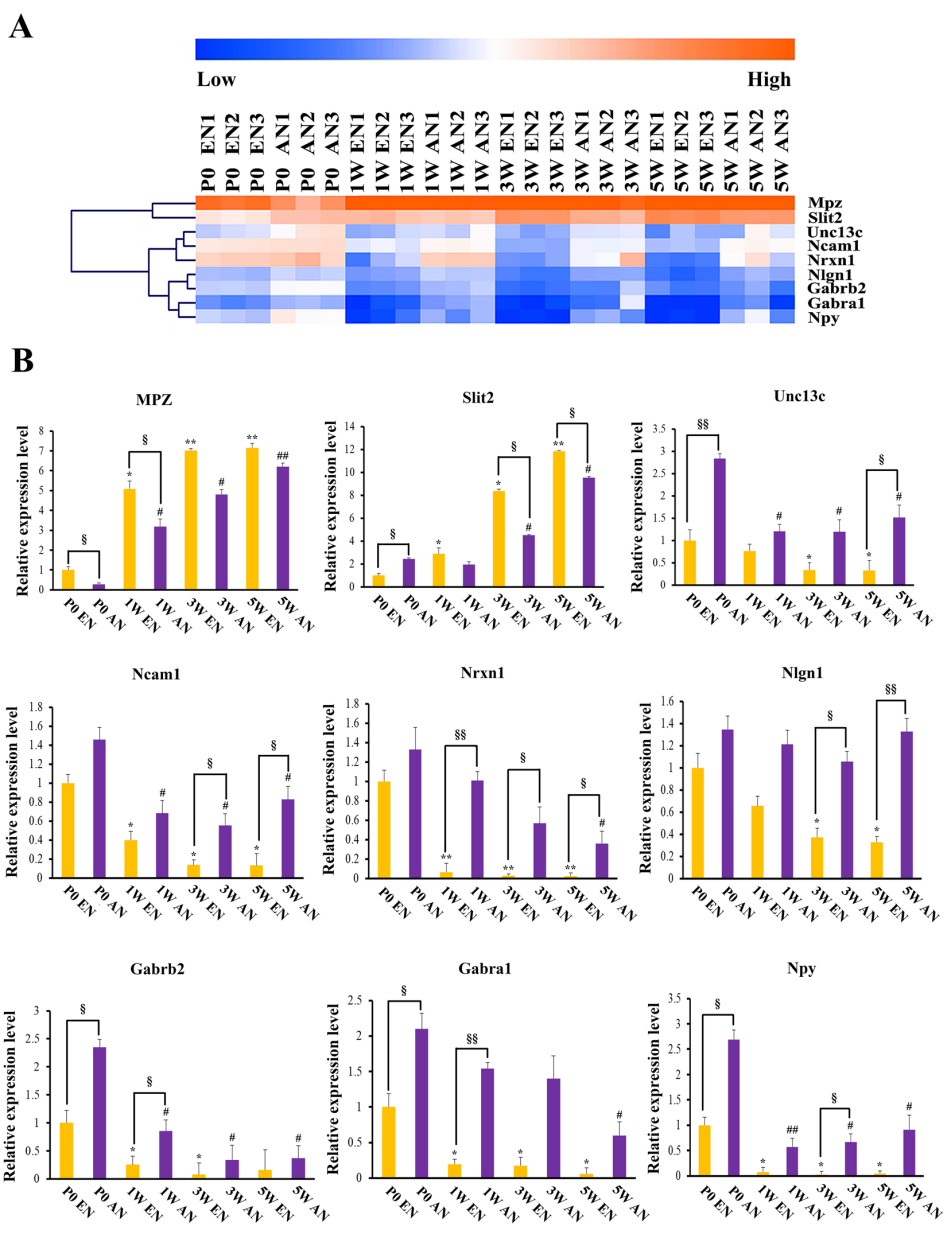
Real-time quantitative polymerase chain reaction-based validation of key genes associated with efferent and afferent nerve fibre development. Heat map clustering displaying the relative mRNA expression levels of 9 differentially expressed genes selected from all 24 samples (8 groups in triplicate). The orange colour indicates the genes that are upregulated, and the blue colour indicates the genes that are downregulated. The values were calculated using comparative 2^−ΔΔCt^ method, with *Gapdh* levels as the internal control (efferent nerve fibres are shown in yellow and afferent nerve fibres are shown in purple). Error bars represent standard deviations from the mean. Data were obtained in three independent experiments. ^*, #,§^*p* < 0.05; ^**, ##, §§^*p* < 0.01; ^*^in efferent nerve fibre development, ^#^in afferent nerve fibre development, ^§^difference between efferent and afferent nerve fibre expression.

Several genes were considered representative regulatory genes. These genes were selected from the microarray analysis data for RT-qPCR validation. They included *Mpz*, *Slit2*, *Unc13c*, *Ncam1*, *Nrxn1*, *Nlgn1*, *Gabrb2*, *Gabra1*, and *Npy*. The heat map in Fig. 8A shows the expression pattern for each selected gene in all 24 samples (8 groups in triplicate). The RT-qPCR results are summarized in the histograms (Fig. 8B). *Slit2* mRNA expression in afferent nerve fibres was significantly up-regulated when compared with that in efferent nerve fibres at P0, although this trend was reversed thereafter. *Nrxn1* mRNA expression in afferent nerve fibres was significantly up-regulated when compared with that in efferent nerve fibres at P0. Interestingly, this differential expression pattern was even more obvious than that for *Slit2* because *Nrxn1* mRNA expression levels were decreased during development in efferent nerve fibres. Coincidentally, the expression patterns of *Nlgn1*, *Gabrb2*, and *Gabra1* mRNA were similar to that of *Nrxn1*. These similarities indicated that the above genes may have essential roles in motor and sensory nerve fibre development. The RT-qPCR results were consistent with the data from the microarray analysis.

## Discussion

Under normal physiological circumstances, neurons maintain proper functioning condition by transporting subcellular components to growth cone terminals via axoplasmic flow. However, when the continuity of axons is completely disrupted, the distal stump of the nerve trunk initiates a process known as Wallerian degeneration^22,23^. When injured neurons have achieved homoeostasis after trauma-induced disruptions, the intrinsic neuronal regenerative capacity is activated to prompt the proximal stump of the nerve trunk to initiate the regenerative programme. The key neuronal element responsible for axonal regeneration is the growth cone, which is a specialized structure in both motor and sensory neurons^24,25^. Generally, growth cone navigation for achieving synaptic rearrangement exhibits a molecular correlation with neuronal differentiation/maturation^26^, indicating that the regenerative procedure may be fundamentally similar to the process of axon sprouting in the developing neural system^2^.

Peripheral motor and sensory nerves have distinct functions that contribute to the maintenance of normal physiological activities, especially their unique abilities to accurately and selectively reinnervate terminal nerve pathways. Recent studies have demonstrated that Schwann cells obtained from different anatomical contexts can express distinct motor and sensory phenotypes in cell culture systems^27^. However, other evidences have indicated that it is the artificial culture condition that altered the gene expression patterns, and resulted in the distinct phenotypic characteristics of differentially expressed genes^28^. In addition, isobaric tags for relative and absolute quantitation-based analysis of total protein levels in different types of nerves in a quantitative proteomics approach revealed that different motor and sensory nerves have distinct protein expression profiles in naive rats^29^. In the *in vivo* regeneration model, the growth factor expression profiles between denervated motor and sensory nerves had obvious differences, further indicating that Schwann cells express distinct motor and sensory phenotypes that specifically benefit motor and sensory axonal regeneration, respectively^11,30^.

In order to decipher the cellular and molecular events during motor and sensory neuron development, microarray analysis was conducted to identify differential gene expression patterns in both efferent and afferent nerve fibres during different developmental processes. Considering the genders of patients who have peripheral nerve injury in the clinic, both sexes of the C57BL/6 mice were used equally across the analyses. In this study, the efferent nerve fibres mainly comprise axons of motor neurons whose cell bodies are located in the ventral horn of the spinal cord, while, the afferent nerve fibres mainly comprise the central branches of axons extending from DRG neurons that are biochemically and anatomically comparable to the efferent nerve fibres. In the peripheral nerve fibres, myelinating Schwann cells inevitably remain in the microarray samples collected using conventional dissecting methods^31,32^, which may also play important roles in peripheral neural development and regeneration. We will further discuss these phenomena with respect to the involvement of Schwann cells as well.

Summarizing the GO analysis results, genes involved in axon guidance, cell differentiation, and GABAA receptor activity subcategories are significantly differentially expressed at all the four time points examined, indicating that the fundamental molecular and cellular mechanisms underlying the regulation of motor and sensory nerve differentiation/maturation may be biologically distinct. Commissural axons grow along stereotyped dorsoventral trajectories in the early developing spinal cord. The projection patterns are encoded by the spatiotemporal distributions of axon guidance cues, specifically netrin-1/ DCC signalling for ventral attraction and SLIT/ROBO signalling for dorsal repulsion^33^. The SLIT proteins are best known for their classic roles in mediating chemorepulsion through the ROBO receptors during axon guidance^34^. However, accumulating evidence indicates that the functional repertoire of SLIT proteins and ROBO also plays essential roles in neurogenesis, stem cell regulation, and organ development^35^. It has been reported that SLIT2 contributes to branching/arborisation of sensory trigeminal axons in the developing mammalian CNS^36^. Intriguingly, several groups have reported that SLIT1 is only expressed in peripheral neurons, while other SLIT family members, such as SLIT2 and its receptors ROBO1 and ROBO2 are also expressed in Schwann cells^37^, and SLIT2 functions as a repellent in cultured Schwann cells via Ras homologue gene family member A-myosin signalling pathway^38^. In this study, *Slit2* mRNA expression was found to be higher in sensory nerve fibres than in motor nerve fibres at the P0 developmental stage. This is consistent with previous reports describing *Slit* expression patterns in the embryonic spinal cord. Intriguingly, this developmental characteristic changes after 1 W. The *Slit2* mRNA expression level was dramatically up-regulated in motor nerve fibres from 1 W to 5 W, so that its expression in motor nerve fibres was significantly higher than that in sensory nerve fibres at the 3 W and 5 W developmental stages. The differential expression patterns of SLIT proteins in efferent and afferent nerve fibres led to our speculation regarding the specific regulatory role of SLIT/ROBO signalling in motor and sensory neuronal differentiation/maturation across different developmental stages in mice.

Trans-synaptic interactions involving neuroligin-neurexin complexes are also implicated in the organization of excitatory glutamatergic and inhibitory GABAergic synapses, and play fundamental roles in the regulation of synaptic cell adhesion^39^. Recent studies have indicated that glutamate plays an essential role in sensory input transduction, especially in the nociceptive afferent signalling pathways. Peripheral neuropathic pain and the involvement of inflammatory processes could be attenuated by pharmacologic manipulation of the glutamatergic system^40^. Neurexins constitute a family of polymorphic cell-surface proteins with hundreds of alternatively spliced isoforms that are essential to the synapse architecture. Recent studies have indicated that distinct spliced isoforms of neurexins are selectively expressed in particular neurons, implying that each type of neuron may have a unique neurexin expression pattern^41^. Notably, α-neurexins are not strictly limited in their function as synaptic terminal-specific proteins but are also involved in axon-Schwann cell and perineurial fibroblast interactions^42^. In addition, contactin-associated protein (CASPR)/paranodin, which is a vertebrate homolog of neurexin IV, has been reported to localize at the paranodal region of the Ranvier nodes; CASPR/paranodin links neuronal membrane components with the axonal cytoskeletal network and Schwann cells^43^. In this study, neurexin 1 was found to be significantly differentially expressed between efferent and afferent nerve fibres in the postnatal developmental stages from 1 W to 5 W, suggesting that neurexin 1 may serve as a central hub gene involved in modulating postnatal sensory neuron development. It is noteworthy that neurexin 1 is predominantly expressed both in motor and sensory axons at P0, implying that neurexin 1 plays fundamental roles in embryonic neural development. Neuroligins are thought to be necessary for maintaining synapse integrity, and function through recruitment of GABARs and ionotropic glutamate receptors. The microarray results indicated that neuroligin 1 mRNA expression level gradually decreased as motor neuron development proceeded, and that the significant differential expression between motor and sensory neurons was only detected at relatively mature stages (postnatal 3 W and 5 W). These data suggested that neuroligin 1 may follow a different modulating mechanism than its trans-synaptic partner neurexin 1 in regulating motor and sensory neuron development.

The principal inhibitory neurotransmitter, GABA, is broadly distributed in both CNS and PNS. Accumulating evidence indicates that the formation of GABAergic synapses is crucial for the formation and maintenance of stereotypic neural networks during CNS development^44^. Moreover, recent evidence has confirmed that GABAergic inputs occur in the mouse embryonic spinal cord as early as at the embryonic age of 12.5 days, implying that GABAergic functions may be essential at very early developmental stages in mice^45^. The GABA-induced activities are mediated via its receptors, chloride-permeable ionotropic GABAA and GABAC receptors, as well as G-protein-coupled metabotropic GABAB receptors^46^. Interestingly, accumulating evidence demonstrates that the myelin-producing Schwann cells also express GABAA and GABAB receptors during neural development and regeneration, and that GABAA and GABAB receptors cross-interact with neuroactive steroids^47,48^ via activation of Src and phospho-focal adhesion kinase signalling^49^. The GABAB receptors are involved in myelination in Schwann cells but not in their proliferation^50^, and GABAB receptors are fundamental in regulating Schwann cell maturation and small nociceptive C-fibres^51,52^. The microarray data in this study indicated that *Gabra1* and *Gabrb2* levels were dramatically decreased in motor and sensory nerve fibres after 1 W. This was especially obvious in motor neuron development, consistent with previous reports that GABAergic activity contributes to the early stages of CNS development. Moreover, *Gabra1* and *Gabrb2* were significantly differentially expressed only at relatively early developmental time points (P0 and 1 W). To complicate matters more, other GABAR subtypes have been shown to be implicated in neuronal differentiation/maturation events in the following developmental windows (3 W and 5 W), further indicating that GABA and its various receptors have substantial roles in determining motor and sensory neuronal cell fate specification across development in mice.

The current findings reveal fascinating aspects of differential gene expression signatures between peripheral efferent and afferent nerve fibres at different developmental stages in mice. The precise and balanced trans-synaptic cell adhesion system is crucial for the establishment of functioning neuronal circuits. Thus, accurate reinnervation of motor and sensory original targets requires appropriate synaptic plasticity, which is needed to fine-tune neural development and regeneration. Generally, the physiological and molecular mechanisms that modulate peripheral nerve regeneration are most intensively studied in the nerve injury model. These data serve as valuable resources, and provide a new perspective regarding the possible cellular and molecular mechanisms underlying motor and sensory development. Further in-depth studies are needed to identify the hub genes and key molecular events that regulate peripheral motor and sensory neuronal cell fate determination.

## Methods

### Separation of Efferent and Afferent Nerve Fibres

C57BL/6 mice at different developmental stages (P0, 1 W, 3 W, and 5 W) were supplied by the Experimental Animal Center of Nantong University. All animal protocols were approved by the Committee of Nantong University, and were performed in accordance with the guidelines of the Administration Committee of Experimental Animals, Jiangsu Province, China.

The animals (n = 9 for each developmental time point) were sacrificed using an overdose of CO_2_ by inhalation. The lumbar vertebral canal was opened, and the spinal cord was exposed. After the spinal arachnoid was carefully peeled off under a dissecting microscope, the efferent and afferent nerve fibres arising from spinal cord segments L4–L6 were dissected and separately collected into 8 tubes containing ice-cold D-Hank’s solution (Gibco, Carlsbad, CA, USA) corresponding to the 8 groups (four time points and two types of nerve fibres). Tissues were then cut into small pieces (no larger than approximately 1 mm^3^) and transferred into liquid nitrogen, where they were stored until further processing.

### RNA Isolation and Microarray Hybridization

Samples were electronically homogenized on ice and then centrifuged at 13,200 rpm for 15 minutes at 4 °C. The resulting supernatant was collected and used in the following steps. Total RNA was isolated using mirVana RNA Isolation Kit (Ambion, Austin, TX, USA) according to the product manual. After the total RNA was purified using the RNeasy Mini Kit (Qiagen, Valencia, CA, USA; product number [p/n]: 74104), its quality was determined based on RNA integrity number (RIN) obtained using a bioanalyzer 2100 system (Agilent Technologies, Santa Clara, CA, USA). Samples with RIN values ≥7 were used for further procedures.

The cDNA was labelled using Cyanine-3-CTP Quick Amp Labeling Kit (Agilent Technologies, p/n: 5190–2305). Agilent SurePrint G3 Mouse GE V2.0 (8 × 60 K, Design ID: 074809) microarrays (Agilent Technologies) were hybridized using the Agilent Gene Expression Hybridization Kit (Agilent Technologies, p/n: 5188–5242). The arrays were then scanned using an Agilent Scanner G2505C (Agilent Technologies). Agilent feature-extraction software (version 10.7.1.1) was used to analyse the microarray images and obtain raw data. Further, GeneSpring GX software (version 12.5) was used to complete the basic analysis. The raw data were normalized using the quantile algorithm. The R software (version 2.13.0) was used for deeper analysis of the microarray data. The limma (linear regression model) package was used to statistically analyse differentially expressed genes.

The microarray analysis was performed by Shanghai OEBiotech Technology Co., Ltd. (Shanghai, China). For each group, data from three independent experiments were assessed to ensure reproducibility. All data (raw and normalized data) was Minimum Information About a Microarray Experiment-compliant, and has been deposited to the National Center for Biotechnology Information database (accession number: GSE113820). Genes and pathways with *p* value < 0.05 and a mean expression fold change greater than 2 were considered statistically significant.

### Bioinformatic Analyses

#### Principal Component Analysis, Z-scores, and Hierarchical Clustering

We performed PCA using the “Population PCA” tool on the Harvard Medical School webpage. The Z-scores (standard scores) were calculated and hierarchical clustering was performed based on log2-transformed mean-centred datasets, as described previously^17^. Hierarchical clustering analysis was performed using Multi Experiment Viewer software (version 4.9). In addition, the STEM software (version 1.3.11) was used to visualize the enrichment analysis of the temporal expression profiles of differentially expressed genes.

#### Gene Ontology Analysis

The differentially expressed genes were classified using the following GO categories: Biological Processes, Cellular Components, and Molecular Functions. Database for Annotation, Visualization, and Integrated Discovery (DAVID) bioinformatic resources were used for further analysis of GO category enrichment^53^, and the expression profiles for each GO subcategory were calculated as previously described^17,23^.

#### Kyoto Encyclopedia of Genes and Genomes Pathway

The KEGG bioinformatics database was integrated with DAVID tools, and used to systematically screen differentially expressed genes, essentially as previously described^23^.

#### Ingenuity Pathway Analysis

The IPA (Ingenuity Systems; www.ingenuity.com; Redwood City, CA, USA) is an online software package used to identify canonical pathways and gene networks, and to further categorise specific physiological processes. The Ingenuity Pathway Knowledge Base was used for deep analysis of the global molecular network, and revealed interactions among the differentially expressed genes.

### Real-Time Quantitative Reverse Transcription Polymerase Chain Reaction

We performed RT-qPCR analysis of the mRNA expression levels of the differentially expressed genes identified in the microarray analysis, including *Mpz*, *Slit2*, *Unc13c*, *Ncam1*, *Nrxn1*, *Nlgn1*, *Gabrb2*, *Gabra1*, and *Npy*, to validate the microarray results. A total amount of 0.5 μg RNA from the peripheral nerve fibre samples was used as a template to perform RT-qPCR. The reverse-transcribed cDNA was synthesized using the Prime-Script Reagent Kit (Takara, Dalian, China); PCR was performed using SYBR Green Premix Ex Taq (Takara) on the StepOne Real-Time PCR System (Applied Biosystems) in triplicate for each sample. The relative mRNA level was calculated using the comparative 2^−ΔΔCt^ method (normalized to glyceraldehyde 3-phosphate dehydrogenase mRNA [*Gapdh*] levels). The sequences of the primer pairs are listed in Table S1.

### Statistical Analysis

All quantitative data are presented as mean ± standard deviation. The data were analysed using SPSS 11.5 software (Chicago, IL, USA). Statistical analysis was performed using one-way analysis of variance, followed by Scheffe’s post hoc t-tests; *p* values <0.05 were considered statistically significant.

## Electronic supplementary material

Dataset 1

Dataset 2

Dataset 3
